# Association between socioeconomic indicators and pulse wave velocity (PWV) measurements in population studies: a systematic review and meta-analysis

**DOI:** 10.1186/s12889-025-23094-4

**Published:** 2025-05-26

**Authors:** Galatia Photiou, Panayiotis Kouis, Zoi Papasavva, Demosthenes B. Panagiotakos, Nicos Middleton, Andrie G. Panayiotou

**Affiliations:** 1 Cardiovascular Epidemiology and Genetics Laboratory, Department of Rehabilitation Sciences, School of Health Sciences, Cyprus University of Technology, 95 Irinis street, Limassol, 3041 Cyprus; 2 Respiratory Physiology Laboratory, Medical School, University of Cyprus, Nicosia, Cyprus; 3 Department of Nutrition and Dietetics, Harokopio University, Athens, Greece; 4 Department of Nursing, School of Health Sciences, Cyprus University of Technology, Limassol, Cyprus

**Keywords:** Arterial stiffness, Pulse wave velocity, socioeconomic status, Socioeconomic indicators, Education, Income

## Abstract

**Background:**

Arterial stiffness, which is estimated by pulse wave velocity (PWV), has been associated with cardiovascular morbidity and mortality; however, there is sparse literature on socioeconomic (SES) indicators and PWV.

**Methods:**

In this systematic review and meta-analysis, evidence was retrieved from cross-sectional and cohort studies on the association of SES indicators (both single and composite) with arterial stiffness. A total of 16,331 records were reviewed and 9 studies included.

**Results:**

Low educational level was associated with an increase of 0.26 m/s in PWV (95% CI: 0.15 to 0.37; *p* < 0.001; I^2^ = 78.2%), whereas low SES was associated with a 0.32 m/s increase of PWV (95%CI: 0.09 to 0.56, *p* = 0.008, I^2^ = 93.6%).

**Conclusion:**

Results underscore the importance of socioeconomic position as a determinant of arterial stiffness. Future studies could benefit from longitudinal designs and more homogeneous SES measures, as well as considering composite indicators of SES like neighborhood/area environment and characteristics, along with individual indicators.

**Supplementary Information:**

The online version contains supplementary material available at 10.1186/s12889-025-23094-4.

## Introduction

Arterial Stiffness of the central arteries is likely to increase with age because elastin becomes replaced by less compliant collagen. As a result of arterial stiffening, the arteries lose their capacity to function as a physiological buffer during cardiac pulsation and relaxation [1–3]. When arteries lose their elasticity, the speed of pulse waves is increased and they arrive back to the heart earlier, during systole, which leads to an increased load on the heart [4]. Arterial stiffness has been associated with several conventional cardiometabolic risk factors and cardiovascular morbidity [5–7]. In addition, it has been shown to be an independent predictor of all-cause and cardiovascular mortality [8]. Pulse wave velocity (PWV) is the most used surrogate measure of arterial stiffness, representing the speed at which pressure waves travel along the aorta and large arteries. Pulse wave velocity (PWV) is the gold standard for noninvasive arterial stiffness assessment, calculated by dividing the distance between two arterial sites by the pressure wave transit time [9]. The foot-to-foot method, which measures the time difference between pulse waveforms, is the most widely used technique for estimating transit time [10]. Among various PWV types, carotid-femoral PWV (cfPWV) is the most validated for predicting cardiovascular events and mortality, serving as the benchmark for central arterial stiffness [11].Brachial-ankle PWV (baPWV), which measures stiffness from the brachial to the ankle arteries, is also used clinically but may be influenced by peripheral arterial conditions, potentially affecting its specificity for central arterial stiffness. Aortic PWV is measured between the aortic root and descending thoracic aorta [11]. Arterial pulse waveforms required for PWV measurement can be obtained using methods such as applanation tonometry, echocardiography, and cardiac magnetic resonance imaging [9]. A faster PWV indicates increased arterial stiffness, making it a simple, non-invasive, and reproducible clinical too [12]. Recently, a method for predicting PWV using age and blood pressure information, without direct arterial measurements, devoid of a measurement tool, was developed and validated [13]. Numerous studies have confirmed that estimated PWV (ePWV) is a robust predictor of patients’ cardiovascular risk [14–17]. Since ePWV can be acquired through a simple calculation without specialized equipment, it holds high clinical utility but may lack the precision of cfPWV and baPWV. While all three methods aid in cardiovascular risk stratification, cfPWV remains the most reliable indicator of central arterial stiffness and prognosis [13].

Pulse wave velocity (PWV) is closely associated with the severity and chronicity of hypertension (HTN). Arterial stiffness is both a consequence and a contributor to hypertension, creating a vicious cycle that accelerates cardiovascular (CV) complications. Hypertension aggravates arterial stiffness through increased mechanical stress on the arterial wall, leading to structural remodeling, collagen deposition, and elastin degradation. Conversely, increased arterial stiffness leads to reduced arterial compliance, which exacerbates hypertension by impairing the cushioning function of large arteries, thereby elevating systolic blood pressure (BP) and pulse pressure [10, 18].

In addition to hypertension, several vascular risk factors, including smoking, diabetes mellitus, and atherosclerosis, contribute to arterial stiffness. Smoking accelerates arterial stiffening through oxidative stress and endothelial dysfunction, while diabetes promotes non-enzymatic glycation of arterial wall proteins, further reducing elasticity [19]. Atherosclerosis, characterized by lipid deposition and plaque formation, exacerbates arterial stiffness by reducing arterial distensibility and increasing PWV. The cumulative effect of these factors results in progressive vascular aging, further compounding the burden of hypertension and cardiovascular risk [20].

Moreover, arterial stiffness has been extensively studied for its association with coronary artery calcification (CAC), a marker of subclinical atherosclerosis. This relationship underscores the systemic nature of arterial calcification and its contribution to cardiovascular risk. Elevated cfPWV has been positively associated with higher CAC scores, indicating a greater burden of subclinical atherosclerosis. For instance, a study involving 1,172 participants found that increased cfPWV correlates with an elevated risk of higher CAC scores, suggesting its utility in identifying individuals at risk for subclinical atherosclerosis [21] but also higher values of baPWV has been linked to an increased prevalence of CAC. In a study of 1,196 Japanese men, elevated baPWV was associated with a higher prevalence of CAC, highlighting the potential of baPWV as a non-invasive marker for coronary artery disease [22].

Arterial stiffness is not merely a marker of vascular aging but also an independent predictor of adverse cardiovascular events. Increased arterial stiffness directly contributes to CV morbidity and mortality by aggravating systolic BP and widening pulse pressure. This occurs through the acceleration of pulse wave reflection, which causes the reflected wave to return to the central aorta during systole rather than diastole, thereby increasing systolic BP and imposing additional strain on the left ventricle [23]. This heightened systolic overload leads to left ventricular hypertrophy, reduced coronary perfusion, and an increased risk of heart failure, myocardial infarction, and stroke [3].

Moreover, widened pulse pressure due to arterial stiffness is a strong predictor of cardiovascular risk, particularly in elderly individuals. The chronic elevation of systolic BP contributes to endothelial dysfunction, arterial remodelling, and plaque instability, which collectively increase the likelihood of adverse events such as ischemic stroke and coronary artery disease [24]. Given these associations, arterial stiffness assessment through PWV measurement provides valuable prognostic information and highlights the need for early intervention to mitigate CV risk in hypertensive individuals.

In 1967, the Whitehall studies, were the first to document an inverse association between socioeconomic status (SES) and mortality [25] Later studies have reported higher PWV in American adolescents [26] with socioeconomic disadvantage as well as lower educational achievement [27], while male Japanese civil servants that were less educated were also shown to have higher brachial–ankle PWV [28]. There is also some evidence that neighborhood physical and social environments are related to several risk factors for poor cardiovascular health [29], with residents from neighborhoods with lower SES, having both a higher burden of cardiovascular disease and a higher incidence and mortality of CVD [30–32].

Currently, there is sparse evidence in the literature regarding the association between composite and individual socioeconomic indicators, such as education, individual/family income and neighborhood deprivation, and higher arterial stiffness using PWV, a validated surrogate marker of arterial stiffness. In this study, we aimed to systematically review published scientific literature reporting on commonly used socioeconomic indicators, both individual and composite, at the individual/family and neighborhood level and arterial stiffness as estimated with PWV.

## Materials & methods

### Search strategy

Two electronic databases, PubMed and EMBASE were searched from inception until December 2024 using algorithms that consisted of keywords, medical subject terms, and relevant combinations. All algorithms were adjusted as appropriate for each electronic database and are presented in supporting information (see Suppl. File 1). Search words for SES included several different terms used to describe SES in the literature such as, “social class“[MeSH Terms], “socioeconomic status”, “educational status“[MeSH Terms], while also trying to capture neighborhood socioeconomic position (residence characteristics“[MeSH Terms], “neighborhood status” etc). The search was carried out by two independent reviewers (G. P. & Z. P.) who screened titles and abstracts to identify eligible studies for full-text evaluation. During the full‐text evaluation, the reference lists of the evaluated studies were also screened for additional eligible studies. Final selection was based on full‐text evaluation, and any disagreements between the two reviewers were resolved independently by two senior researchers (A.G.P., P.K.).

The systematic review adhered to the guidelines of the Preferred Reporting Items for Systematic Reviews and Meta-Analyses (PRISMA) and has been registered with the International Prospective Register of Systematic Reviews (ID: CRD42023415253).

### Studies’ selection

#### Inclusion criteria

This review focuses on cross-sectional and cohort studies as these made up the majority of studies on the topic. All studies were eligible for inclusion if they were conducted in adults (> 18 years old), reporting current or subsequent surrogate indices of vascular ageing (PWV) and any predetermined indicators of socioeconomic status (SES), composite or individual, such as education, income (personal/family), social class (personal/family), employment grade, as well as neighborhood or neighborhood deprivation characteristics (such as physical, social, and economic attributes of a community in the form of housing characteristics, crime rates or access to healthcare or recreational spaces), in any part of the study (relationship addressed as a main or secondary aim of the included study).

#### Exclusion criteria

The electronic search was limited to articles in the English language. Exclusion criteria included studies that were performed on animals, infants, children, and adolescents. Lack of data on either SES indicators or PWV also resulted in exclusion. Studies looking at air pollution and arterial stiffness were also excluded as, although air pollution is often correlated with SES, it is primarily an environmental determinant of vascular health, and thus not the focus of this study.

### Data extraction

The primary outcomes of interest were the socioeconomic indicators and arterial stiffness (PWV). After the initial screening, extraction of data from all eligible studies, was performed independently by G.P. and Z.P. using a standardized data extraction form which included the following variables: Author, year, sample size, country, sex, age (years), exposure examined (e.g. SES, education, social class, neighborhood characteristics/deprivation), exposures definitions/comparisons, arterial stiffness outcomes assessed, analysis/comparison method (adjustments/etc.) and findings.

### Quality assessment

The methodological quality of the included studies was assessed independently by two researchers (G.P. and A.G.P.) using the Newcastle-Ottawa Scale (NOS), which rates quality of observational studies [33]. The NOS adapted for non-randomized studies (cohorts) was used for cohort studies and an adapted version was used for studies with a cross-sectional study design. The criteria included in the two NOS tools used in this study are described in detail in Suppl. File 1. Briefly, the NOS uses a “star system” in which a study is rated on three broad perspectives according to different criteria. For cohort studies these are: (1) Selection (representativeness of the exposed cohort, selection of the non-exposed cohort, ascertainment of exposure, demonstration that outcome of interest was not present at start of study), (2) Comparability (comparability of cohorts on the basis of the design or analysis) and (3) Outcome (assessment of outcome, was follow-up long enough for outcomes to occur, adequacy of follow up of cohorts). For cross-sectional studies, the same broad perspectives apply but specific criteria are different: (1) Selection (representativeness of the sample, sample size, non-responders, ascertainment of the exposure-risk factor), (2) Comparability (comparability of subjects in different outcome groups on the basis of design or analysis and confounding factors controlled) and (3) Outcome (assessment of outcome, statistical test). Studies could get a total of 4 points for selection, 2 for comparability, and 3 for assessment of the outcome or exposure for a total of 9 points per study. Thus, when assessing individual studies, a maximum of nine stars could be obtained. Study quality is assessed on the following star scale: 0 − 2 (poor quality), 3 − 5 (fair quality), 6 − 9 (good/high quality).

### Qualitative and quantitative synthesis

The main study characteristics were summarized in table format with particular emphasis on the socio-economic indicators used across the retrieved literature by each study. In addition, the association between the examined socio-economic indicators with PWV levels is presented in the form of the distribution of PWV across the different strata of the socio-economic indicators or crude or adjusted effect measures where available. Studies included in the qualitative synthesis are also narratively described in the text. For the purposes of quantitative synthesis (meta-analysis), a subset of the retrieved studies was considered suitable, based on the homogeneity of the employed study design and the socio-economic indicators examined. Furthermore, the meta-analysis relied on the actual measurement of PWV across the socio-economic strata which was available from most included studies. Reported effect measures were not synthesized as these were largely derived using different statistical methodologies adjusting for different confounders in each study. As a result, the quantitative synthesis relied on a random-effects generalized inverse variance analysis of the standardized mean difference (SMD) to evaluate the differences in PWV levels between the different strata (low Vs high) of the socio-economic indicators, focusing on education and SES as these were defined in each study. In case of additional strata of education and SES were reported in the original studies, the meta-analysis focused on the comparison between the highest and the lowest stratum. A-priori sensitivity analyses were planned to assess the robustness of pooled estimates and investigate the impact of studies characterized by lower quality assessment score or substantially different methodology. Finaly, heterogeneity in the summary estimates was assessed with the I^2^ statistic which ranges between 0 and 100% and describes the overall proportion of the variation in the effect estimate that can be attributed to between-studies heterogeneity. To explore heterogeneity in the meta-analysis for education, two post-hoc separate subgroup analyses were carried out. The first excluded small sample size studies and the second excluded studies where the education level assessed was other than the individual’s. To explore heterogeneity in the meta-analysis for SES, a post-hoc subgroup analysis synthesizing only studies reporting associations for family/household SES rather than personal SES was carried out. All statistical analysis was carried out using STATA (Version 18, StataCorp LLC).

## Results

### Literature search results

In total, 17,370 records were retrieved from the two electronic databases searched, i.e. PubMed and EMBASE. The title and abstract of all records underwent initial screening based on the inclusion and exclusion criteria. Of the initial 17,122 records, 27 were sought for retrieval and were eligible for full-text assessment. After full-text assessment 7 of them were further excluded from the analysis as they were assessing the association of arterial stiffness as measured by AIx/PP/AP/baPWV/cfPWV with ambient air pollution and residential outdoor exposure without any socioeconomic indicators. A further 10 were excluded because they did not report on a relevant exposure and outcome, as set in the eligibility criteria.

**Fig. 1:**
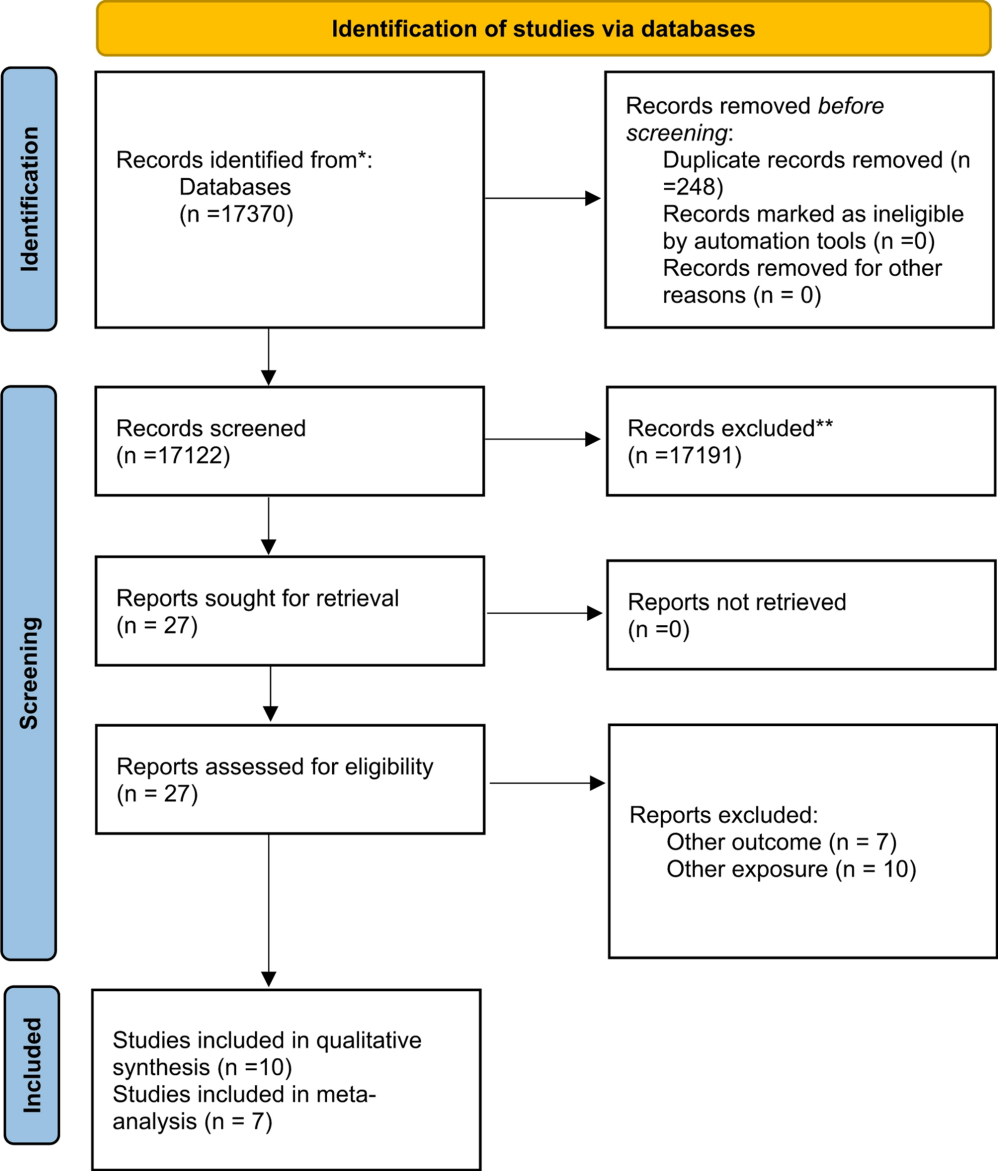
PRISMA 2020 flow diagram for new systematic reviews which included searches of databases and registers only *From*: Page MJ, McKenzie JE, Bossuyt PM, Boutron I, Hoffmann TC, Mulrow CD, et al. The PRISMA 2020 statement: an updated guideline for reporting systematic reviews. BMJ 2021;372:n71. doi: 10.1136/bmj.n71 For more information, visit: http://www.prisma-statement.org/

Finally, a total of ten (10) studies were included in the qualitative synthesis and seven (7) studies included in relevant meta-analyses. The PRISMA diagram summarizing the systematic review process can be seen in Fig. 1.

### Study characteristics

A total of 44,308 participants were included in the 10 selected studies. In these studies, arterial stiffness was assessed using Pulse Wave Velocity (PWV), whereas socioeconomic indicators included education, income, poverty status, employment status, father’s social class as well as neighborhood characteristics and neighborhood deprivation index. Of the included studies, 7 were cross-sectional studies, and 3 were longitudinal cohort studies. Of the 3 longitudinal cohort studies, two (Trudel et al. and Kähönen et al.) [34, 35] also provided data on cross-sectional comparisons across exposure groups. As shown in Table 1, five studies were conducted in the USA (*n* = 5), two in Finland (*n* = 2), and the remaining three in the UK, Korea and Brazil. Spikes et al., and Islam JI et al. [36, 37], reported on data from cohort studies in the USA (ARIC and JHS and MECA cohort studies respectively). The two studies conducted in Finland used data from the Young Finns Study and Coelho DM et al., and Trudel X et al., reported on data from the ELSA-Brazil multicenter prospective study and from the Whitehall II longitudinal study respectively. The study by Kim HL et al. reported data from the Korea National Health and Nutrition Examination Survey (KNHANES) [38]. In all studies, participation of males ranged from 35.3 to 73.2% and the mean age of the study populations ranged from 41.21 to 75 years. Data collection relied on previous medical records, questionnaires, and clinical visits for assessment of PWV.

**Table 1:** Characteristics and results of included studies

#	Study	Study Design	Sample (*n*) (Country)	Gender (male)	Age (years)*	Exposures Definitions/Comparisons	PWV measurements (crude, used in meta-analysis)	Analysis & Comparison methods	Findings
1	Logan JG et al. [42]	Cross-sectional study	102 (USA, Korean Americans)	41 (40%)	39.6 ± 9.9	**Education (binary)** Low Education (*n* = 33): Community college or less High Education (*n* = 69): University or higher	cfPWV* Low Education: 7.49 ± 1.06 High Education: 6.76 ± 1.10	ANOVA (no adjustments)	**cfPWV (mean ± SD)** Low Education: 7.49 ± 1.06 High Education: 6.76 ± 1.10
2	Wendel CR et al. [43]	Cross-sectional study	2270 (USA)	999 (44%)	47.7 ± 9.3	**SES (Binary**, **poverty status)**: Above poverty line (61.3%) Below poverty line (38.7%) Education (continuous): Years of education	cfPWV* African Americans: Low SES: 7.81 ± 1.96 High SES: 8.49 ± 3.24 Whites: Low SES: 7.85 ± 1.93 High SES: 7.89 ± 2.65	Hierarchical Multiple Regression Models (adjusted for age, sex, race, alcohol, SES, education, WRAT-3, smoking, illicit drugs, CES-D, BMI, total cholesterol, lipid lowering medications, hypertension, diabetes, CVD)	**cfPWV (** ***β*** **(SE))** Low SES: −0.252 (0.224) Education: −0.062 (0.023)†
3	Trudel X.et al. [35]	Longitudinal cohort study (with baseline cross-sectional comparison)	5242 (UK, Civil servants)	3836 (73.2%)	65.5 ± 5.8	**Father’s social class: (categorical)** I–II IIIn–IIIm IV–V **Employment grade: (categorical)** Administrative Professional/executive Clerical/support **Household income: (categorical)** £50– >100 000 £25–49 999 <£9999–24 999 **Education: (categorical)** BA/BSc and higher degree Advanced level No academic/ordinary level	cfPWV* (baseline comparisons) Father’s social class: I-II: 8.5 IIIn–IIIm: +0.15 IV–V: +0.21 Employment grade: Administrative: 8.6 Executive: +0.02 Support: +0.13 Household income: £50– >100 000: 8.6 £25–49 999: +0.05 <£9999–24 999: +0.11 Education: BA/BSc & higher: 8.6 Advanced: +0.05 No academic: +0.10	Linear mixed models. (adjustment for age, sex, ethnic group, and mean arterial pressure at time of measurement, BMI, smoking, and alcohol intake. SBP, heart rate, total cholesterol, HDL cholesterol, diabetes, antihypertensive medication)	**cfPWV (Increase(95%CI))** Father’s social class: 0.16 (-0.16, 0.49) Employment grade: 0.38 (0.11, 0.65)† Household income: 0.58 (0.32, 0.85)† Education: 0.30 (0.01, 0.58)†
4	Islam JI et al. [38]	Cross-sectional study	JHS (*n* = 1582) (USA, Black Americans) MECA (*n* = 451) (USA, Black Americans)	JHS: 559 (35.3%) MECA: 175 (38.9%)	JHS: 53.2 ± 10.2 MECA: 53.1 ± 10.2	**Neighborhood characteristics: (categorical)** JHS: Social cohesion Problems Violence MECA: Social Cohesion Activity with neighbors Aesthetic quality Walking environment Food access Violence Safety	JHS– cfPWV* Social cohesion Low cohesion: 8.18 ± 6.64 High cohesion: 7.65 ± 6.02 Problems Low problems:7.46 ± 5.68 High problems: 8.55 ± 7.11 Violence Low violence:7.29 ± 5.54 High violence: 8.68 ± 7.13 MECA– cfPWV* Social Cohesion Low cohesion: 7.67 ± 2.29 High cohesion: 7.55 ± 1.87 Violence Low violence:7.64 ± 2.12 High violence: 7.59 ± 2.07	Multivariable Linear Regression Models (adjustment for age, sex, SBP, BMI, diabetes. smoking status, annual household income)	**JHS - cfPWV (** ***β*** **(CI))** Social cohesion: -0.32 (-0.63, -0.02)† Problems: -0.33 (-0.64, -0.02)† Violence: -0.30 (-0.61, 0.002) **MECA - cfPWV (** ***β*** **(CI))** Social cohesion: -0.06 (-0.24, 0.12) Activity neighbors: -0.23(-0.4,-0.05)† Aesthetic quality: 0.08 (-0.09, 0.25) Walking environment:0.07 (-0.09, 0.24) Food access: 0.03 (-0.15, 0.20) Violence: -0.03(-0.21, 0.15) Safety: 0.04 (-0.13, 0.21)
5	DuBose LE et al. [41]	Cross-sectional study	113 (USA)	56 (49%)	67.3 ± 0.7	Education (binary): > HS (*n* = 77): high school (HS) ≥ 13 years of formal education: at least one year of college or greater ≤ HS(*n* = 36): high school (HS) ≤ 12 years of formal education: HS diploma or less	cfPWV* > HS: 10.3 ± 0.2 ≤ HS: 10.5 ± 0.4	Two tailed Student t test (unadjusted)	cfPWV* > HS: 10.3 ± 0.2 ≤ HS: 10.5 ± 0.4
6	Puolakka E. et al. [44]	Longitudinal cohort study	2566 (Finland)	1176 (46%)	Women: 10.50 ± 4.97 Men: 10.44 ± 5.07	**Income (categorical)**: Annual income strata were determined on an 8-point scale: at the time of enrollment (childhood) from 1 (< 2500€) to 8 (> 16800€) and in adulthood from 1 (< 10 000€) to 8 (> 70000€).	Not available	Linear Regression for the association of childhood SES on PWV in adulthood (adjusted for age at baseline, sex, BMI, SBP, and vegetable consumption, SES in adulthood)	**cfPWV (*β*(SE))** − 0.048 ± 0.023†
7	Coelho DM et al. [40]	Cross-sectional study	13,365 (Brazil)	6108 (45.7%)	51.0** (45.0–58.0)	**Maternal education (categorical)**: High school Complete elementary school Incomplete elementary school Never attended school	cfPWV*** Whites Never: 9.4 (9.28, 9.54) High School: 9.15 (9.08, 9.21) Browns Never: 9.57 (9.47, 9.71) High School: 9.05 (8.92, 9.18) Blacks Never: 9.76 (9.6, 9.9) High School: 9.2 (8.96, 9.43)	Linear Regression (adjusted for sex and age, smoker, physical activity, current body weight, height, mean arterial pressure, heart rate, antihypertensive medication, diabetes and participants’ own education)	**cfPWV (** ***β*** **(CI))** Whites: High school: Reference category Complete elementary school: -0.06 (-0.15 to 0.03) Incomplete elementary school: 0.05 (-0.03 to 0.13) Never attended school: 0.01 (-0.12 to 0.15) Browns: High school: Reference category Complete elementary school: -0.09 (-0.24 to 0.06) Incomplete elementary school: 0.08 (-0.05 to 0.21) Never attended school: 0.18 (0.01 to 0.34)† Blacks: High school: Reference category Complete elementary school: 0.24 (-0.01 to 0.49) Incomplete elementary school: 0.35 (0.13 to 0.57)† Never attended school: 0.44 (0.18 to 0.70) †
8	Kähönen, E. et al. [36]	Longitudinal cohort study (with baseline cross-sectional comparison)	1761 Finland	790 (44.9%)	11.2 ± 4.5	**Parental SES (categorical)**: > 0.5 SD (high) ≥ 0 to 0.5 SD −0,5 to < 0 SD <−0.5 SD (low) **Adulthood individual SES (categorical)**: > 0.5 SD (high) ≥ 0 to 0.5 SD −0,5 to < 0 SD <−0.5 SD (low) **Neighborhood deprivation (categorical)**: <−0.5 SD (low) < 0.0 to − 0.5 SD 0.0. to 0.5 SD > 0.5 SD (high)	cfPWV* Adulthood individual SES Low SES: 9.75 ± 1.7 High SES: 8.6 ± 1.7	Frequencies & proportions (categorical variables) or means and standard deviations (continuous variables) for low, low intermediate, high intermediate, and high cumulative neighborhood deprivation groups. A random coefficient generalized mixed model was used for the analysis of mean differences. (adjustment for age, sex, place of birth and adulthood individual SES and neighbourhood deprivation (for parental SES analysis), or parental SES and neighbourhood deprivation (for adulthood individual SES analysis) or parental and adulthood individual SES (for neighbourhood deprivation analysis))	**cfPWV (mean difference (95% CI))** Parental SES: > 0.5 SD (high): 0.00(reference) ≥ 0 to 0.5 SD: 0.22 (-0.03-0.47) −0,5 to < 0 SD: 0.14 (-0.11-0.40) <−0.5 SD (low): 0.22 (-0.07-0.51) Adulthood individual SES: > 0.5 SD (high): 0.00 (reference) ≥ 0 to 0.5 SD: 0.19 (-0.07-0.45) −0,5 to < 0 SD: 0.34 (0.08–0.59) † <−0.5 SD (low): 0.54 (0.23–0.84) † Neighborhood deprivation: <−0.5 SD (low): 0.00(reference) < 0.0 to − 0.5 SD: 0.36 (0.13–0.59) 0.0. to 0.5 SD: 0.31 (0.06–0.57) † > 0.5 SD (high): 0.37 (0.05–0.70) †
9	Spikes T.A et al. [37]	Cross-sectional study	3362 USA	1203 (36%)	75 ± 4.9	**Education (categorical)**: < than High School (HS) HS (HS/GED) Some College and above (Vocational school and college) Postgraduate (Graduate school) Total combined family income: <$25,000 $25,000-<$50,000 ≥$50,000	cfPWV* Education African Americans < HS: 13 ± 3.7 HS:12.4 ± 3.5 Some college:12.3 ± 3.5 Postgraduate: 11.4 ± 3.2 Whites < HS: 12.1 ± 3.2 HS:12 ± 4.6 Some college:11.4 ± 3.4 Postgraduate: 11.1 ± 3.0 Total combined family income African Americans <$25,000: 13 ± 3.7 $25,000-<$50,000: 12 ± 3.2 ≥$50,000: 12 ± 3.2 Whites <$25,000: 12.2 ± 4.3 $25,000-<$50,000: 12 ± 3.7 ≥$50,000: 11.1 ± 3.8	Multivariable Linear Regression Models (adjustment for age, site, sex, race, HDL, total cholesterol, BMI, smoking, alcohol, lipid lowering medications, diabetes, SBP and antihypertensive medication)	**cfPWV (β (CI))** Education (all) < HS (Ref) HS: 0.03 (-0.40 to 0.47) ≥Some college: -0.05 (-0.49 to 0.39) Post-graduate: -0.45 (-0.95 to 0.04) Total combined family income (all): <$25,000 (ref) $25,000-<$50,000: -0.45 (-0.78 to -0.11)† ≥$50,000: -0.53 (-0.88 to -0.19)† Education (African Americans) < HS (Ref) HS: -0.62 (-1.28 to 0.05) ≥Some college: -0.26 (-0.89 to 0.38) Post-graduate: -0.97 (-1.65 to 0.29)† Total combined family income (African Americans): <$25,000 (ref) $25,000-<$50,000: -0.69 (-1.26 to -0.13)† ≥$50,000: -0.57 (-0.15 to 0.01) Education (Whites) < HS (Ref) HS: 0.39 (-0.19 to 0.97) ≥Some college: 0.25 (-0.35 to 0.84) Post-graduate: -0.06 (-0.76 to 0.63)† Total combined family income (Whites): <$25,000 (ref) $25,000-<$50,000: -0.35 (-0.77 to 0.07)† ≥$50,000: -0.48 (-0.90 to 0.05)
10	HL Kim et al. [39]	Cross-sectional study	13,539 (Korea)	5815 (42.9%)	52.9 ± 16.7	**Individual income**: Lowest, second, third, highest level **Household income**: Lowest, second, third, highest level **Education level**: College graduation or higher High school graduation Middle school graduation No schooling or elementary school only	**ePWV** Individual income: Lowest: 8.84 Highest: 8.74 Household income: Lowest: 10.69 Highest: 7.89 Education level No or elementary: 11.36 College or higher: 7.58	Multivariate linear regression analyses showing the associations of ePWV with household income and education level (adjustment for age, sex, BMI, SBP, glycated hemoglobin, HDL, glomerular filtration rate and uric acid) Multivariate binary logistic linear regression analyses with abnormal outcome set as ePWV ≥ 8.47 m/s (adjustment forage, sex, BMI, hypertension, diabetes mellitus, dyslipidemia, cigarette smoking, glomerular filtration rate and uric acid)	**ePWV (β (SE))** Household income -0.055 (0.011)† Education level -0.076 (0.013)† **ePWV (OR (95%CI))** Household income Reference: Highest Second: 1.32 (1.13, 1.55)† Third: 1.99 (1.69, 2.34)† Lowest:3.13 (2.58, 3.79)† Education level Reference: College/higher High school: 1.34 (1.17, 1.54)† Middle school: 3.17 (2.55, 3.95)† No/elementary: 11.42 (8.68, 15.02)†

JHS: Jackson Heart Study; MECA: Morehouse-Emory Cardiovascular Center for Health Equity; SES: Socioeconomic status; PWV (m/s): Pulse Wave Velocity; cfPWV: Carotid-femoral pulse wave velocity; SD: Standard Deviation; SE: Standard Error; **MECA**: Morehouse-Emory Cardiovascular Center for Health Equity study; JHS: Jackson Heart Study; WRAT-3: Wide Range Achievement Test 3; CES-D: Center for Epidemiologic Studies Depression Scale; BMI: Body Mass Index; CVD: cardiovascular disease; HDL: high-density lipoprotein; SBP: Systolic Blood Pressure

*Mean and standard deviation; **Median and Interquartile Range *** Mean and 95% Confidence Interval †: Statistically significant at the *p* < 0.05 level

Of the 10 included studies, the majority (*n* = 8) had reported on educational level, with 6 of them on individual education and 2 on maternal or paternal education. Two studies reported on neighborhood characteristics or neighborhood deprivation, 6 of them on income and 2 on employment class or grade. All studies (*n* = 10) were included in the narrative review.

#### Meta-analysis

Among the 10 studies included in the qualitative synthesis, a total of 7 studies [34–36, 39–42] provided data for quantitative synthesis and based on the availability of data, it was decided to run two individual quantitative meta-analyses. The first meta-analysis included studies with data on educational level (low vs. high as defined by each study) and cfPWV (5 studies, *n* = 8 populations) [34, 36, 39–41], and the second on socioeconomic status (income/poverty) (low vs. high as defined by each study) and cfPWV (4 studies, *n* = 6 populations) [34–36, 42]. The meta-analyses included only studies employing a cross-sectional study or reported data on cross-sectional comparisons. A corresponding meta-analysis including data from longitudinal studies was not possible due to scarcity of data.

### Association of educational level and socioeconomic status with cfPWV

#### Educational level (low vs. high)

**Fig. 2:**
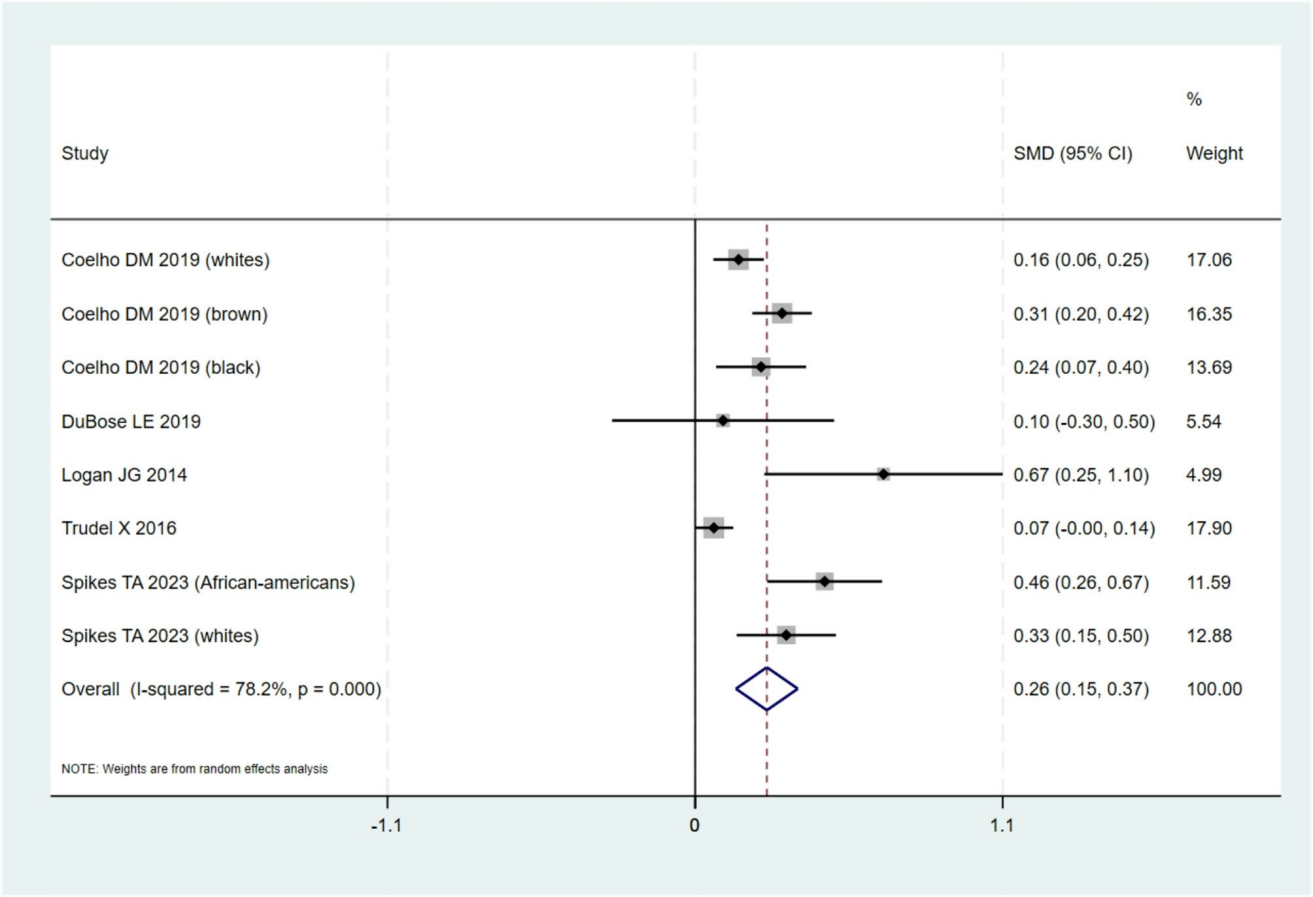
Pooled standardized mean difference of cfPWV in Low vs. High Education

As can be seen in Fig. 2, when comparing the pooled standardized mean difference of PWV in Low vs. High Education groups, having a low educational level was associated with an increase of 0.26 of PWV (95%CI: 0.15 to 0.37; *p* < 0.001; I^2^ = 78.2%). All studies were consistent, with the same direction of effect as shown in the forest plot. When excluding the study with the lowest score on the qualitative assessment (Dubose et al.), the sub-analysis resulted in a similar association between low educational level and PWV, with low educational level being associated with an increase of 0.27 of PWV (95%CI: 0.15 to 0.38, *p* < 0.001, I^2^ = 81.2%). In post-hoc, subgroup analyses, aiming to explore the high heterogeneity observed, exclusion of small sample size studies [40, 41]as well as exclusion of the study by Coelho et al. [39] which reported association with PWV for maternal education, did not result in any notable changes in either the pooled estimate or the I^2^. Furthermore, from the included studies that addressed the effect of Education on PWV, only 2 [40, 41], did not report any adjusted analyses for the outcome of interest. The remaining 3 studies [34, 36, 39], reported additionally adjusted results for several appropriate confounders. These included, age, sex, BMI, blood pressure and a number of common cardiovascular risk factors such as cholesterol, smoking, diabetes and antihypertensive medication. As can be seen in detail in Table 1, the reported adjusted associations between Low Vs High Education and PWV were in the same direction in all included studies and remained statistically significant in the fully-adjusted models in the study by Kim et al. (β: -0.076, SE: 0.013) and the non-white populations in the studies by Spike et al. (high Vs low in African-Americans: *β*: -0.97,95%CI: -1.65, -0.29) and Coelho et al. (low Vs high maternal education in Browns: *β*: 0.18, 95%CI: 0.01, 0.34, and Blacks: *β*: 95%CI: 0.44; 0.18, 0.70). Similarly, the longitudinal comparison (PWV increase over 5 years) reported by Trudel X et al. demonstrated that PWV increase was more profound among those with lower education status (*β*: 0.30, 95% 0.01, 0.58) compared to those with higher education status [34].

#### Socioeconomic status (low vs. high)

**Fig. 3:**
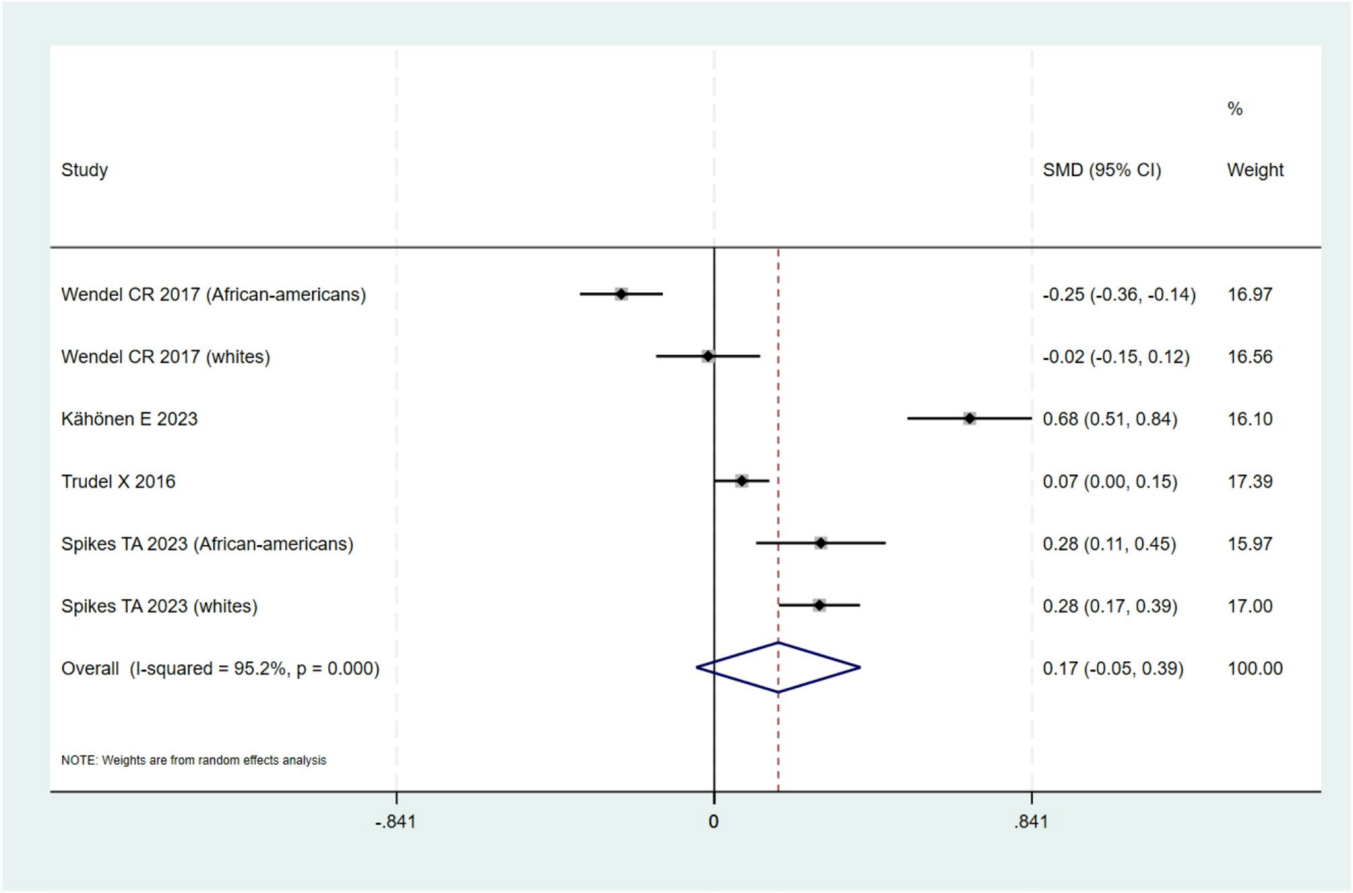
Pooled standardized mean difference of cfPWV in Low vs. High SES

For the exposure of SES, 6 populations were included in the meta-analysis (Fig. 3) [34–36, 42]. Although all included studies were reporting on SES (low vs. high), there was some variation in the way SES was estimated with 2 studies (3 populations) including family/household income measurements [34, 36], 1 study (2 populations) using the poverty line cut-off [42]and 1 using a composite indicator that included income, education and employment [35]. Low Vs high SES (as defined in each study/population) was associated with a 0.17 increase of PWV, however results were not statistically significant (95% CI: -0.05 to 0.39, *p* = 0.127) probably due to high heterogeneity between the included studies (I^2^ = 95.2% suggesting substantial to considerable heterogeneity). It is of note that most studies had a similar direction of effect, supporting an association between lower SES and higher PWV, with only one study reporting a different direction of effect. This could be perhaps explained by the definition of low Vs high SES, which in this particular study was defined as below or above the USA poverty line. When excluding this study (Wendel et al.) from the meta-analysis, low SES was associated with a statistically significant 0.32 increase of PWV (95%CI: 0.09 to 0.56, *p* = 0.008, I^2^ = 93.6%). Additionally, in a post-hoc, subgroup analysis, synthesizing only studies reporting associations for family/household SES [34, 36] rather than personal, result in only modest reduction in heterogeneity as assessed by I^2^ (SMD: 0.20, 95%CI: 0.04, 0.36, *p* = 0.002, I^2^ = 83.5%.

For the cross-sectional association between SES and cfPWV, all included studies [34, 35, 38, 42] reported adjusted results. Fully adjusted models included different combinations of age, sex and a combination of: smoking, BMI, illicit drug use, heart rate, alcohol, cholesterol, diabetes, blood pressure, lipid lowering and antihypertensive medication, place of birth, neighborhood deprivation index and father’s social class. As presented in Table 1, the reported adjusted associations between Low Vs High SES and cfPWV were all in the same direction and remained statistically significant only in three studies that had used family/household income as a measure of SES [34, 36, 38]. Specifically, the fully adjusted reported associations (Low Vs high SES) between family/personal income and cfPWV were *β*: -0.055, SE:0.011 and *β*: -0.53; -0.88, -0.19 (High Vs Low) for the study by Spikes et al. In addition, the longitudinal study by Puolakka et al. [43] which assessed the impact of childhood SES on adulthood PWV demonstrated a statistically inverse relationship (β: -0.062 m/s ± standard error: 0.022), with those exhibiting a higher SES in childhood, being characterized by lower PWV in adulthood. Similarly, the longitudinal comparison (PWV increase over 5 years) reported by Trudel X et al. demonstrated that PWV increase was more profound among those with lower SES (*β*: 0.58, 95% 0.32, 0.85) compared to those with higher SES status [34].

#### Neighborhood environment and cfPWV

Only two studies reported on PWV and neighborhood deprivation/neighborhood characteristics. One addressed a cross-sectional comparison and the other a longitudinal, while the other used a combined neighborhood deprivation index [35] while the other report associations between individual neighborhood characteristics and PWV [37]. For these reasons, a meta-analysis was not carried out. Nevertheless, the study by Islam SJ et al. [41], reported that certain neighborhood characteristics, such as lower socioeconomic status, higher crime rates, and fewer resources (e.g., access to healthcare, recreational spaces), were associated with increased arterial stiffness among Black adults. In the same direction, the second study, a prospective study by Kähönen E. et al. [35], reported that individuals who lived in deprived neighborhoods during their whole lifetime had a higher risk of developing arterial stiffness as adults.

### Quality assessment results

The results of the quality assessment are shown in Table 2. Given that the nature of exposure, i.e. education/SES, is usually self-reported, studies were given fewer stars for Selection, resulting in three studies scoring a total of nine stars and another 3 scoring a total of eight stars. Of the remaining studies, one scored 7 stars, another 6 stars and two scored 5 stars, thus all studies included in the analysis were deemed to be of at least good/high quality as per the NOS.

**Table 2:** Newcastle-Ottawa quality assessment scale adapted for non-randomized studies (cohorts) and the version for cross-sectional studies

Study (first author)	Study design	Selection				Comparability	Outcome			Total score
		Representativeness of the sample	Sample size	Nonrespondents	Ascertainment of exposure	Based on design and analysis	Assessment of outcome	Statistical test		
Logan et al.	Cross-sectional	**	**-**	**-**	**-**	**	**	*		7*
Wendel et al.	Cross-sectional	*	*	*	*	**	**	*		9*
DuBose LE et al.	Cross-sectional	*	**-**	**-**	*	**-**	**	*		5*
Spikes T.A et al.	Cross-sectional	*	**-**	**-**	**-**	**	**	*		6*
Coelho DM et al.	Cross-sectional	*	*	**-**	*	**	**	*		8*
HL Kim et al.	Cross-sectional	*	**-**	**-**	*	**		*		5*
Islam JI et al.	Cross-sectional	*	*	**-**	*	**	**	*		8*
		**Selection**				**Comparability**	**Outcome**			
		**Representativeness of the exposed cohort**	**Selection of the non-exposed cohort**	**Ascertainment of exposure**	**Demonstration that outcome of interest was not present at start of study**	**Comparability of cohorts on the basis of the design or analysis**	**Assessment of outcome**	**Was follow-up long enough for outcomes to occur**	**Adequacy of follow up of cohorts**	
Trudel X.et al.	Cohort	*	*	*	*	**	*	*	*	9*
Puolakka et al.	Cohort	*	*	-	*	**	*	*	*	8*
Kähönen et al.	Cohort	*	*	*	*	**	*	*	*	9*

## Discussion

In this systematic review and meta-analysis, evidence was retrieved from cross-sectional and cohort studies on the effect of socioeconomic indicators (individual and composite) on arterial stiffness as estimated by PWV and published from inception until December 2024 in the English language. Education was the most frequently used socioeconomic indicator across the included studies, with 7 out of 9 studies having assessed educational level. In the meta-analysis performed, the SMD in PWV between low and high education level was 0.26, with consistent results amongst the studies included. This finding further supports the previously reported association between higher educational levels and better health outcomes, including cardiovascular health [44].

This association could be explained by several mechanisms as education is frequently associated with higher health literacy, enabling individuals to make more informed health-related decisions. Those with higher education levels are more likely to engage in healthier lifestyles, such as maintaining a balanced diet, engaging in regular physical activity, avoiding smoking, and managing stress effectively. Additionally, higher educational attainment often leads to better employment opportunities and higher income, which can provide access to better healthcare resources and environments conducive to maintaining cardiovascular health [45, 46].

When looking at the association between socioeconomic status (SES), as assessed mainly by income indicators, and PWV and including all relevant studies, there was a non-statistically significant association between lower SES and PWV. This non-statistically significant finding may suggest that the relationship between SES and arterial stiffness is complex and may not be directly influenced by SES alone, especially as measured mainly by income. It is of note, that the included studies had high heterogeneity, with associations ranging from positive to negative, even in similar populations (for example the 2 included studies from the USA with African-American populations). This may in part be attributed to the way income was categorized in different studies with studies comparing income above/below poverty line vs. high/low income. Unfortunately, we could not account for this variability in income categorization as effect estimates in included studies were only reported based on their own study categorization. However, when removing the study using the USA poverty line as the cut-off between low and high SES, low SES was associated with a statistically significant 0.32 increase of PWV (95%CI: 0.09 to 0.56) in the remaining studies.

This study is not without limitations. Searches were limited to studies published in the English language allowing for the possibility that relevant studies published in non-English journals were not included. However, a wide definition of SES was used in the search, allowing for both composite and individual SES indicators. It is of note that most of the included studies in the meta-analysis were from the USA with additional studies in the UK, Finland and Brasil. Given the complex nature of SES in addition to cultural relevance of included SES indicators, caution should be exercised when generalizing these findings in other populations. This limitation is further highlighted by the fact that due to the small number of studies included, we could not further examine possible differences by region or gender. Aggregate estimates of studies were used in the analysis (vs. individual patient data), thus issues with original studies remain; however, the quality of included studies was assessed based on specific criteria and all studies had an overall good quality. Finaly, despite most individual studies controlling for appropriate confounders and reporting adjusted effect estimates for the relationship of SES and/or education with PWV, we only synthesized the difference in actual PWV measurements between different exposure strata. Although this approach did not consider potential confounders, it avoids several inhomogeneity concerns as adjusted effect estimates were derived using different statistical methodologies adjusting for different confounders in each study. In addition, the narrative synthesis of the adjusted effect estimates largely confirms a similar direction in the relationship between SES and/or education with PWV as the one derived using the meta-analysis of the actual measurements.

As SES is a complex notion that encompasses income, education, occupation, and social status, and further influenced by the cultural and overall landscape of individual countries/populations, it is important that future studies take this into account with more populations studied. While higher SES often correlates with better health outcomes, the direct impact of SES or specific SES attributes on PWV may be mitigated by other factors such as genetic predispositions, lifestyle choices, and psychosocial factors, as individuals with similar SES might still experience different levels of stress, access to healthcare, and environmental exposures, all of which can influence PWV [47–52].

In this meta-analysis, we report a significant association between low vs. high educational level as well as low vs. high SES and arterial stiffness as estimated by cfPWV. We were not able to synthesize quantitatively the effect of neighborhood characteristics and PWV as only two studies reported on neighborhood environment, with only one using a neighborhood deprivation index and one reporting a cross-sectional association and the other one longitudinal association. However, both studies reported a positive association between the worse neighborhood environment and PWV, thus emphasizing the critical role of neighborhood environment on cardiovascular health and highlighting the need for improving social determinants of health, such as neighborhood conditions to reduce the burden of cardiovascular diseases. A high heterogeneity is also reported between studies in the way that SES is both measured and used, with income and education being the most commonly used proxy for SES. Studies looking on the association between socioeconomic characteristics both at the individual and the neighborhood/area level could benefit from longitudinal designs and further homogeneity in measurements of SES, to better understand its impact on PWV and overall cardiovascular health.

An increase in aortic cfPWV by 1 m/s has been previously reported to correspond to an age-, sex-, and risk factor-adjusted risk increase of 14%, 15%, and 15% in total CV events, CV mortality, and all-cause mortality, respectively [19]. A more recent meta-analysis further supports the predictive value of arterial stiffness, especially in patients with higher disease risk for total CVD events and CVD mortality [53]. Carotid-femoral pulse wave velocity is now considered a useful biomarker in improving prediction of CV risk and identifying high-risk populations who may benefit from aggressive CV risk factor management, although limitations in its implementation in clinical practice still persist [54]. We report a SMD of 0.26 of PWV in low vs. high education groups and a SMD of 0.32 for low vs. high SES highlighting the importance of socioeconomic status on arterial health and subsequent CV risk. While confidence intervals around the pooled mean differences were large, indicating the low accuracy and high heterogeneity of included studies, the effects were consistent -especially in the case of educational level, perhaps due to the higher homogeneity of measuring education in different studies/populations.

In conclusion, while our study underscores the importance of socioeconomic measures and especially educational attainment in influencing arterial stiffness and health, as reflected in PWV values, it also highlights the need for further research on the complex interplay between SES indicators and cardiovascular health, especially moving forward towards the neighborhood social environment. Future studies could benefit from longitudinal designs, more homogeneous measures of SES to better understand its impact on PWV and overall cardiovascular health, as well as considering the neighborhood/area environment and characteristics in addition to individual SES measures.

## Electronic supplementary material

Below is the link to the electronic supplementary material.

Supplementary Material 1

## Data Availability

The datasets used and analyzed during the current study are available from the corresponding author on reasonable request.

## Abbreviations

PWV: Pulse Wave Velocity
SES: Socioeconomic Status
CVD: Cardiovascular Disease
MeSH: Medical Subject Headings
IMT: Intima-Media Thickness
ASI: Aortic Stiffness Index
NOS: Newcastle-Ottawa Scale
SMD: Standardized Mean Difference
AIx: Augmentation Index
PP: Pulse Pressure
AP: Augmentation Pressure
baPWV: Brachial-ankle pulse wave velocity
cfPWV: Carotid-femoral pulse wave velocity
CV: Cardiovascular
JHS: Jackson Heart Study
MECA: Morehouse-Emory Cardiovascular Center for Health Equity
SD: Standard Deviation
SE: Standard Error
