# Supplementary material for: Association between socioeconomic indicators and pulse wave velocity (PWV) measurements in population studies: a systematic review and meta-analysis
Source: BMC Public Health. 2025 May 26;25:1937. doi: 10.1186/s12889-025-23094-4 (PMC12105119; doi:10.1186/s12889-025-23094-4)
Supplement: Supplementary file 1 — Supplementary Material 1 [file 12889_2025_23094_MOESM1_ESM.docx]

**Association between socioeconomic indicators and pulse wave velocity (PWV) measurements in population studies: a systematic review and meta-analysis**

**Supplementary File**

1. **information on search strategy:**

Exposure

Social gradient

Socio economic index

Social inequality

Socioeconomic disparities

Educational level

Educational attainment

Socioeconomic position

Socioeconomic status

(("neighborhood"[All Fields] OR "residence characteristics"[MeSH Terms] OR ("residence"[All Fields] AND "characteristics"[All Fields]) OR "residence characteristics"[All Fields] OR "neighborhood"[All Fields]) AND status[All Fields])

("social class"[MeSH Terms] OR ("social"[All Fields] AND "class"[All Fields]) OR "social class"[All Fields] OR ("socioeconomic"[All Fields] AND "status"[All Fields]) OR "socioeconomic status"[All Fields])

"educational status"[MeSH Terms] OR ("educational"[All Fields] AND "status"[All Fields]) OR "educational status"[All Fields]

Outcome

Pulse Wave Velocity

("Vascular Stiffness"[mh] OR Elasticity[mh] OR "Arterial Pressure"[mh] OR "Vascular Calcification"[mh] OR "Pulse Wave Analysis"[mh] OR "pulsatile flow"[mh] OR ("internal carotid" AND index) OR distensibil*[tiab] OR augmentation OR "stiffness index" OR PWV OR CPP OR "pulsatile flow")

Search Algorithms

Pubmed

(("neighborhood"[All Fields] OR "residence characteristics"[MeSH Terms] OR ("residence"[All Fields] AND "characteristics"[All Fields]) OR "residence characteristics"[All Fields] OR "neighborhood"[All Fields]) AND status[All Fields]) OR ("social class"[MeSH Terms] OR ("social"[All Fields] AND "class"[All Fields]) OR "social class"[All Fields] OR ("socioeconomic"[All Fields] AND "status"[All Fields]) OR "socioeconomic status"[All Fields]) OR ("educational status"[MeSH Terms] OR ("educational"[All Fields] AND "status"[All Fields]) OR "educational status"[All Fields])) AND ("Vascular Stiffness"[mh] OR Elasticity[mh] OR "Arterial Pressure"[mh] OR "Vascular Calcification"[mh] OR "Pulse Wave Analysis"[mh] OR "pulsatile flow"[mh] OR ("internal carotid" AND index) OR distensibil*[tiab] OR augmentation OR "stiffness index" OR PWV OR CPP OR "pulsatile flow")

Embase

(neighborhood OR residence OR “social class” OR “socioeconomic status” OR socioeconomic OR “educational status”) AND (“Vascular Stiffness” OR Elasticity OR “Arterial Pressure” OR “Vascular Calcification” OR “Pulse Wave Analysis” OR “pulsatile flow” OR (“internal carotid” AND index) OR augmentation OR “stiffness index” OR PWV OR CPP OR “pulsatile flow”)

Google scholar

(neighborhood OR residence OR “social class” OR “socioeconomic status” OR socioeconomic OR “educational status”) AND (“Vascular Stiffness” OR Elasticity OR “Arterial Pressure” OR “Vascular Calcification” OR “Pulse Wave Analysis” OR “pulsatile flow” OR (“internal carotid” AND index) OR augmentation OR “stiffness index” OR PWV OR CPP OR “pulsatile flow”)

1. **Description of Newcastle - Ottawa Quality Assessment Scale (NOS) criteria for NOS adapted for non-randomized studies (cohorts) and NOS adapted version for cross-sectional studies**

**Newcastle-Ottawa Quality Assessment Scale for cohort studies**

Note: A study can be awarded a maximum of one star for each numbered item within the Selection and Outcome categories. A maximum of two stars can be given for Comparability

Selection

1) Representativeness of the exposed cohort

a) truly representative of the average _______________ (describe) in the community

b) somewhat representative of the average ______________ in the community

c) selected group of users eg nurses, volunteers

d) no description of the derivation of the cohort

2) Selection of the non-exposed cohort

a) drawn from the same community as the exposed cohort

b) drawn from a different source

c) no description of the derivation of the non-exposed cohort

3) Ascertainment of exposure

a) secure record (eg surgical records)

b) structured interview

c) written self-report

d) no description

4) Demonstration that outcome of interest was not present at start of study

a) yes

b) no

Comparability

1) Comparability of cohorts on the basis of the design or analysis

a) study controls for _____________ (select the most important factor)

b) study controls for any additional factor (This criteria could be modified to indicate specific control for a second important factor.)

Outcome

1) Assessment of outcome

a) independent blind assessment

b) record linkage

c) self-report

d) no description

2) Was follow-up long enough for outcomes to occur

a) yes (select an adequate follow up period for outcome of interest) ****

b) no

3) Adequacy of follow up of cohorts

a) complete follow up - all subjects accounted for ****

b) subjects lost to follow up unlikely to introduce bias - small number lost - > ____ % (select an adequate %) follow up, or description provided of those lost) ****

c) follow up rate < ____% (select an adequate %) and no description of those lost

d) no statement

**Newcastle-Ottawa Quality Assessment Scale adapted for cross-sectional studies**

Selection:

1. Representativeness of the sample:
   1. Truly representative of the average in the target population. * (all subjects or random sampling)
   2. Somewhat representative of the average in the target group. * (non-random sampling)
   3. Selected group of users/convenience sample.
   4. No description of the derivation of the included subjects.
2. Sample size:
   1. Justified and satisfactory (including sample size calculation). *
   2. Not justified.
   3. No information provided
3. Non-respondents:
   1. Proportion of target sample recruited attains pre-specified target or basic summary of non-respondent characteristics in sampling frame recorded. *
   2. Unsatisfactory recruitment rate, no summary data on non-respondents.
   3. No information provided
4. Ascertainment of the exposure (risk factor):
   1. Vaccine records/vaccine registry/clinic registers/hospital records only. **
   2. Parental or personal recall and vaccine/hospital records. *
   3. Parental/personal recall only.

Comparability: (Maximum 2 stars)

1. Comparability of subjects in different outcome groups on the basis of design or analysis. Confounding factors controlled.
   1. Data/ results adjusted for relevant predictors/risk factors/confounders e.g. age, sex, time since vaccination, etc. **
   2. Data/results not adjusted for all relevant confounders/risk factors/information not provided.

Outcome:

1. Assessment of outcome:
   1. Independent blind assessment using objective validated laboratory methods. **
   2. Unblinded assessment using objective validated laboratory methods. **
   3. Used non-standard or non-validated laboratory methods with gold standard. *
   4. No description/non-standard laboratory methods used.
2. Statistical test:
   1. Statistical test used to analyse the data clearly described, appropriate and measures of association presented including confidence intervals and probability level (p value). *
   2. Statistical test not appropriate, not described or incomplete.

Cross-sectional Studies:

Very Good Studies: 9-10 points

Good Studies: 7-8 points

Satisfactory Studies: 5-6 points

Unsatisfactory Studies: 0 to 4 points
